# Outcome of Pediatric Cataract Surgeries in a Tertiary Center in Switzerland

**DOI:** 10.1155/2018/3230489

**Published:** 2018-02-25

**Authors:** Sarah Claudia Ambroz, Marc Töteberg-Harms, James V. M. Hanson, Jens Funk, Daniel Barthelmes, Christina Gerth-Kahlert

**Affiliations:** ^1^University Children's Hospital Zurich, Steinwiesstrasse 75, 8032 Zürich, Switzerland; ^2^Department of Ophthalmology, University Hospital Zurich, Frauenklinikstrasse 24, 8091 Zürich, Switzerland; ^3^Neuroimmunology and Multiple Sclerosis Research, Clinic for Neurology, University Hospital Zurich, Frauenklinikstrasse 26, 8091 Zurich, Switzerland

## Abstract

**Purpose:**

To determine and to analyze the outcome of pediatric cataract surgery.

**Methods:**

A retrospective chart review of individuals aged up to 10 years who underwent cataract surgery between January 1, 2004, and December 31, 2014, at the UniversityHospital Zurich, Switzerland.

**Results:**

63 children (94 affected eyes) with bilateral (68/94) or unilateral (26/94) cataract were identified. Surgery was performed at a median age of 1.5 months (IQR: 1.3–2.6 months) for the aphakic group (45/94) and of 50.7 months (IQR: 38.0–78.4 months) for the IOL group (49/94). At the last follow-up visit (median 31.1 months, IQR: 18.4–50.2 months), visual acuity was better in bilateral than in unilateral cataract cases. Posterior capsular opacification (PCO) was diagnosed in 30.9% of eyes without a significant difference in the IOL and aphakic groups (*p* = 0.12). Aphakic glaucoma was diagnosed in 12/45 eyes at a median of 6.8 months (IQR 2.1–13.3 months) after surgery. Microcornea (5/12) and anterior segment anomalies (8/12) were associated with glaucoma development (*p* < 0.05).

**Conclusion:**

Laterality and timing of surgery influence the outcome of pediatric cataract surgery. PCO was the most frequent postoperative complication. Aphakic glaucoma is often associated with ocular developmental abnormalities and a poor visual outcome.

## 1. Introduction

Congenital cataract is the main reason for preventable blindness in children worldwide, with a prevalence of 3 to 4 per 10,000 children in Europe [1, 2]. Although cataracts in children are mostly congenital, acquired cataracts (e.g., following ocular trauma) are also relatively common [3, 4]. Etiology of congenital or acquired childhood cataract includes ocular abnormalities, ocular trauma, intrauterine infections, associated syndromes, or hereditary causes [5].

The long-term outcome of pediatric cataract surgery depends on multiple factors, for example, age at first presentation of cataract and age at surgery, associated ocular anomalies, and development of aphakic glaucoma [6, 7]. A variety of factors determines the likelihood of a successful functional and morphological outcome after pediatric cataract surgery. According to Wu et al., the treatment of congenital cataract patients is among the most difficult and cost-intensive interventions in ophthalmology [1].

Early detection of cataract in children and rapid referral to a pediatric ophthalmology center in order to evaluate surgical indications are essential for successful management [6]. The appropriate timing of surgery is crucial and should be balanced between the severity of amblyopia and the risk of glaucoma development after cataract surgery [7–9]. The implementation of new surgical techniques, such as 23-gauge vitrectomy, has led to reduced surgical trauma [10]. Pediatric cataract often leads to the development of nystagmus, strabismus, and amblyopia [5, 11]. Therefore, careful postoperative care includes therapy for amblyopia and accurate optical rehabilitation [6]. During the follow-up period, it is critical to detect postoperative complications. These complications can manifest long after the procedure and can be severe and frequent [9]. Development of glaucoma after pediatric cataract surgery is one of the most severe complications [12], which depends on such factors as age at surgery, presence of associated ocular pathologies, and whether or not an intraocular lens is primarily implanted [8, 13].

The aim of this study is to gain a better understanding of the multiple factors that influence the outcome of pediatric cataract surgery. We analyzed the patients' characteristics as well as surgical techniques and their associations with postoperative complications, such as posterior capsular opacification (PCO) and aphakic glaucoma.

## 2. Methods

A retrospective review of medical charts and surgical reports was performed on all children diagnosed with cataract who underwent cataract surgery within the first 10 years of life. Surgery was performed between January 1, 2004, and December 31, 2014, at the Department of Ophthalmology at the UniversityHospital Zurich, Switzerland. The study was approved by the Cantonal Ethics Committee of Zurich (protocol number 2015-0324). All surgical records of the department were reviewed to identify cases. Search strings were “cataract” as diagnosis and “age ≤10 years” at the time of surgery. The results were filtered to exclude all cases that had prior cataract surgery and therefore had an intervention other than cataract surgery during the time of the review. Patients without any follow-up examination between January 1, 2004, and December 31, 2015, were excluded from this analysis.

Data collection included demographic, morphological, and functional data at initial presentation and last follow-up (i.e., gender, age, personal and family history, type of cataract, results of anterior and posterior segment examination, presence of strabismus, nystagmus, best corrected visual acuity (BCVA), and intraocular pressure (IOP)), as well as type and techniques of surgery, postoperative management (i.e., amblyopia therapy), and complications. Exclusion criteria were diagnosis of glaucoma, traumatic globe perforation, or retinal detachment prior to cataract surgery. Visual acuity was assessed according to the age and performance of the patient (e.g., preferential-looking charts at ages up to 6 months, Cardiff charts at ages up to 2 years, Patti Pics charts at ages up to 4 years, or Snellen acuity thereafter) and is presented in Snellen acuity notation. Intraocular pressure was measured by Goldmann applanation tonometry or using the TONO-PEN XL (Haag-Streit, Koeniz, Switzerland) or Perkins tonometer (Haag-Streit, Essex, UK), depending on the age and cooperation of patients during examination. Digital palpation was employed in completely uncooperative children; however, no patient was diagnosed with glaucoma based on digital palpation-based measures of intraocular pressure.

Microcornea was defined as horizontal corneal diameter < 9 mm for newborn children and <10 mm for children >2 years of age. Microphthalmia was defined as an eye with an axial length of <19 mm in a 1-year-old child or <21 mm in an adult [14, 15].

Aphakic glaucoma refers to the glaucoma that occurs after congenital cataract surgery [8]. According to the 9th consensus report of the World Glaucoma Association, a diagnosis of childhood glaucoma should be made if two or more of the following characteristics are present: “(1) IOP >21mmHg, (2) optic disc cupping (a progressive increase in cup-disc ratio, cup-disc asymmetry of ≥0.2 when the optic discs are of similar size, or focal rim narrowing), (3) corneal findings (i.e. Haab's striae, corneal edema, or diameter ≥11mm in newborn, >12mm in child <1 year of age, or >13 mm at any other age), (4) progressive myopia or myopic shift coupled with an increase in ocular dimensions out of keeping with normal growth, and/or (5) a reproducible visual field defect that is consistent with glaucomatous optic neuropathy with no other observable reason for the visual field defect” [16].

The technique used during surgery was adapted according to the age and properties of the lens material. In case of soft lens material, a simple aspiration of the lens material with a bimanual irrigation-aspiration system could be used. In case of harder lens material, either a 25 g vitrectomy probe or a phaco tip was used. In older children (usually of age 2 years and older), an IOL was placed in the bag and a posterior capsulotomy was performed via a pars plana access using a 25 g vitrectomy probe. In younger children, eyes were left aphakic and a posterior capsulotomy was performed using either a bent needle or a 25 g vitrectomy probe using the established paracentesis. The use of nonabsorbable sutures was replaced in 2013 by absorbable vicryl sutures, with the result that suture removal was no longer required.

### 2.1. Statistical Analysis

Statistical analyses were performed using SPSS statistical software versions 22 and 23 for Windows (IBM Corp., Armonk, NY, USA) and Microsoft Excel version 14.0.0 for MAC/Windows (Microsoft Corp., Redmond, WA, USA) and http://www.vassarstats.net/index.html (accessed between November 2, 2017, 8 p.m. and November 5, 2017, 8 p.m.). The Fisher exact test was used for categorical variables. Kaplan-Meier survival analysis was used to calculate time to event with event being glaucoma. Results are expressed as *p* values or median and IQR (due to nonnormal distribution of data to avoid skewing by extremely large or small values). *p* values < 0.05 were considered statistically significant.

## 3. Results

**Figure 1:**
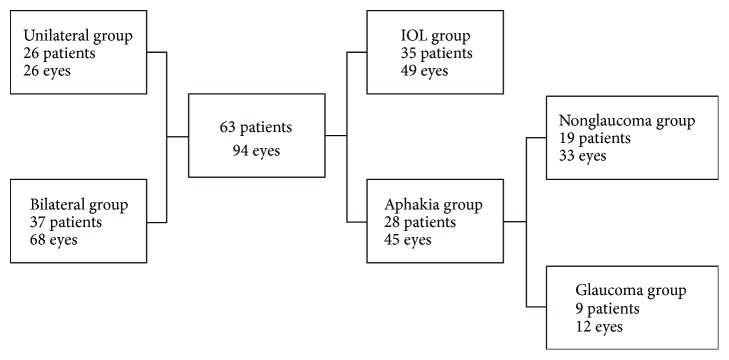
Distribution of 63 patients (94 eyes) included in the investigation.

Medical charts of 69 patients were identified. The following charts were excluded from the study: one patient presented with retinal detachment, one patient with posterior perforation of the globe before surgery, and three patients that suffered from combined congenital glaucoma and cataract. Furthermore, one patient had no documented follow-up visits. Ultimately, the data of 63 children who had surgery during the first 10 years of life were included, which encompasses 94 affected eyes: 35 boys (55.6%) and 28 girls (44.4%) with bilateral (68/94) or unilateral (26/94) cataracts (Figure 1). We included only one eye of six patients with bilateral cataracts due to the second eye undergoing surgery during a time outside of the analyzed period or at a different institution.

### 3.1. Classification of Cataract

Diagnosis of cataract was either congenital or juvenile, with congenital cataract being diagnosed in 82 out of 94 eyes. The diagnosis of juvenile cataract accounted for 12 out of 94 eyes, with 8 instances being acquired cataracts. The details of associated ocular anomalies or secondary causes are listed in Table 1.

### 3.2. First Presentation and Examination

Median age at first presentation of all patients was 15.0 months (IQR: 1.2–50 months), with 18 patients (28.6%) presenting during the first month of life. Ophthalmologists or primary eye centers referred 28 patients (44.4%). The remaining patients were referred by a pediatrician, family physician, a children's hospital (22/63; 13.9%), the parents (3/63), the school physician (1/63), or by the maternity clinic (1/63). In 8/63 cases, no medical chart information regarding the patient's referral was located.

Signs at first presentation were leucocoria (59/94), strabismus (21/94), or nystagmus (9/94). A positive family history of early onset cataracts was documented in seven patients (11.1%).  Table 2 provides an overview of the presenting ocular and systemic anomalies.

### 3.3. Surgery

Surgery was performed at a median age of 22.2 months (IQR: 1.6–50.9 months). An intraocular lens (IOL) was implanted in 49 of 94 eyes (median age 50.7 months at surgery, IQR: 38.0–78.4 months). There were no cases of secondary IOL implantation.

No IOL was implanted in 45 of 94 eyes (median age 1.5 months at surgery, IQR: 1.3–2.6 months). An anterior vitrectomy was performed in 63 eyes (67.0%), and the posterior capsular bag was opened in 81 eyes (86.2%). A microincisional cataract surgery technique (23-gauge) was used in 12 eyes (12.8%) with an IOL implantation in 7/12 eyes. Paracentesis incisions were usually placed in 10 and 2 o'clock positions. Primary posterior capsulotomy was performed in all cases as well as anterior vitrectomy.

**Figure 2:**
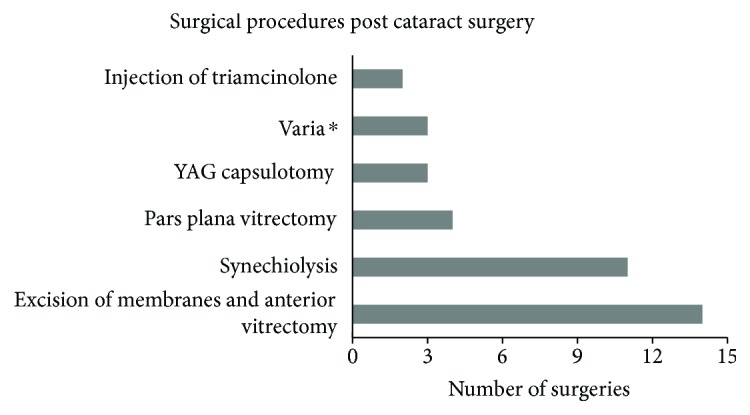
Surgical procedures post cataract surgery (not including glaucoma surgeries, examination under anesthesia, and suture removal). ^∗^Varia (number of surgeries): anterior chamber lavage (1), iridotomy (1), adhesiolysis (1).

Refractive corrections were achieved by contact lenses for near vision, by contact lenses for distance vision with near addition through bifocal or progressive spectacles, or by spectacles only depending on age, development, and parental care. Adverse events due to the use of contact lenses were keratitis and corneal neovascularization (Figure 2). At around two years of age, we implanted IOLs and children received bifocals or progressive glasses. In our study analysis, IOLs were implanted in three children prior to age two years: two of these three children were developmentally delayed and syndromic (although to date not definitively classified). One of the three children received an IOL at 21 months of age.

Amblyopia therapy was recommended in all patients with surgery for unilateral cataract. 37 patients (48 eyes) received therapy for amblyopia. 23/37 of these patients (62.2%) had undergone surgery for unilateral cataract and 14/37 (37.8%) for bilateral cataract. The success rate of amblyopia therapy is limited by compliance and morphological/anatomical factors. Patients who had bilateral cataract surgery and developed an interocular difference in visual acuity or patients who presented with manifest unilateral strabismus as a sign of amblyopia also received patching therapy.

### 3.4. Postoperative Complications

**Figure 3:**
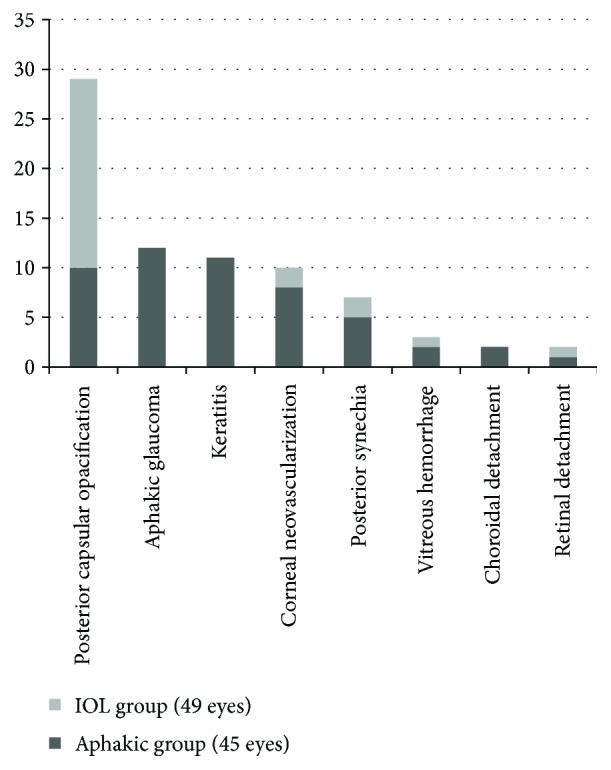
Number of eyes with postoperative complications.

During the follow-up period (median 31.1 months, IQR: 18.4–50.2 months), 52 eyes (55.3%) developed complications, which are summarized in Figure 3. Complete retinal detachment and phthisis in one eye was described in a patient with bilateral cataract who developed complicated aphakic glaucoma requiring several glaucoma surgeries in both eyes. Vitreous hemorrhage and retinal detachment was documented in one patient with Down syndrome at 13.5 months post cataract surgery for traumatic cataract.

PCO occurred in 10/45 eyes (22.2%) in the aphakic group and 19/49 eyes (38.8%) in the IOL group. There was no significant difference in the incidence of PCO in the IOL and aphakic groups (*p* = 0.12). A posterior capsular bag opening and an anterior vitrectomy were performed in 82.8% (24/29 eyes) and 65.5% (19/29 eyes), respectively, of these eyes. In comparison, eyes without documented PCO had a posterior capsular bag opening and an anterior vitrectomy in 87.7% (57/65 eyes) and 67.7% (44/65 eyes), respectively. There was no statistically significant relationship between anterior vitrectomy or opening of the posterior bag and the subsequent development of PCO (*p* = 1.00 and 0.75, resp.). Only 5/13 eyes without opening of the posterior capsule developed PCO, four eyes belonged to the IOL-group and one eye to the aphakic group. There was no statistically significant difference between the IOL and aphakic groups concerning development of PCO in those without opening of the posterior capsular bag (*p* = 0.56).

Secondary surgical interventions were required in 33 eyes (35.1%) including glaucoma surgeries. Examinations under anesthesia and suture removal were not included in the analysis, as plotted in Figure 2. Details of glaucoma surgeries are listed under the section Aphakic Glaucoma.

### 3.5. Functional Outcome

**Figure 4:**
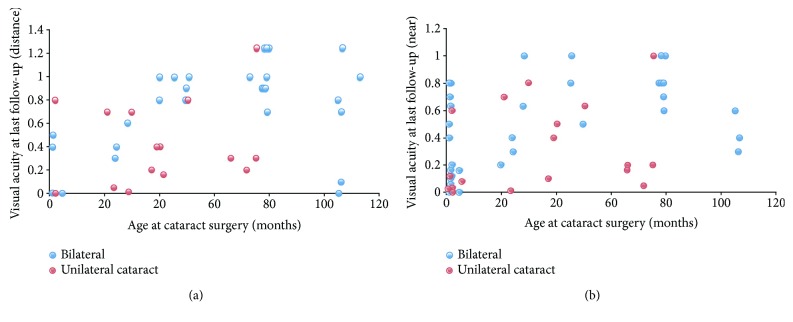
Visual acuity at last follow-up versus age at cataract surgery. Distance (a) and near (b) Snellen visual acuity at last follow-up as a function of age at cataract surgery is plotted for bilateral (blue) and unilateral (red) cataracts. Functional outcome of bilateral cataract appeared better than for unilateral cataract, especially at a later age at surgery.

The median of the last follow-up visit was 31.1 months after cataract surgery (IQR: 18.4–50.2 months) with a median age of 66.8 months (IQR 25.7–99.8 months). Best-corrected visual acuity was greater than or equal to 0.4 decimal Snellen equivalent in 34 eyes for distance and in 36 eyes for near. Snellen acuity equal to or less than 0.2 was documented in 12 eyes for distance and in 21 eyes for near. Visual acuity was better in bilateral than in unilateral cataract cases, especially when surgery was performed at a later age as shown in Figure 4.

There was no significant difference in strabismus development during the follow-up period in the unilateral and bilateral cataract groups (12/37 = 32.4% unilateral versus 7/26 = 27.0% bilateral; *p* = 0.78) nor in the IOL and aphakic groups (10/35 = 28.5% IOL versus 9/28 = 32.1% aphakic; *p* = 0.79). Strabismus was already documented prior to surgery in 12/63 patients (19.0%) and more often in the unilateral (nine patients) than in the bilateral (three patients) cataract group.

Nystagmus was documented preoperatively and persisted during the follow-up period in 1/37 patients (2.7%) with bilateral cataract and 2/26 patients (7.7%) with unilateral cataract. Nystagmus was documented before surgery but not during follow-up in two unilateral and one bilateral cataract patients. Nystagmus developed in four patients during the follow-up period, yet all of these patients had the diagnosis of bilateral aphakic glaucoma. Nystagmus was observed in 10/63 patients (15.9%) at last follow-up (bilateral cataract group 6/37, 16.2%; unilateral cataract group 4/26, 15.4%, *p* = 1.00; IOL group 1/35, 2.9%; aphakic group 9/28, 32.1%, *p* < 0.05). The nystagmus in two of the four patients with unilateral aphakia is not related to the lens status but rather to bilateral macula dragging after severe ROP in one patient and optic disc hypoplasia in the other patient.

### 3.6. Aphakic Glaucoma

Aphakic glaucoma was diagnosed in 12 of 45 eyes (26.7%, 9/28 patients). Median age at first presentation of these patients was 1.0 month (IQR 0.3–2.5 months). Diagnosis of glaucoma was made at a median of 6.8 months (IQR 2.1–13.3 months) after cataract surgery. Kaplan-Meier survival statistics showed a mean survival time of 71.7 ± 7.9 months (95% CI 56.2; 87.2) in the aphakic group. Glaucoma did not develop in the IOL group (Cox survival *p* < 0.001). Children who developed aphakic glaucoma underwent surgery at a median age of 1.5 months (IQR: 1.0–2.4 months), whereas children who did not develop glaucoma had a median age of 30.0 months (IQR: 2.3 months–68.9 months) at the time of surgery. Cataracts in all of these eyes were classified as congenital. A family history of congenital cataract was known in 2/9 patients. Ocular or extraocular anomalies or syndromes were present in 3/9 patients (4/12 eyes) as listed in Table 1. In addition, microcornea (3/12), a flat anterior chamber with a protruding iris and posterior synechiae (1/12), and rubeotic iris with a very small pupil (1/12) were documented in the aphakic glaucoma group. Aphakic glaucoma development was significantly correlated with the clinical signs of microcornea (*p* < 0.05) and anterior segment anomalies (*p* < 0.05). Besides cataract (3/12 eyes), no anterior or posterior segment anomaly was documented.

An anterior vitrectomy and opening of the posterior capsular bag was performed for 10/12 eyes, with 3/12 eyes undergoing the 23-gauge surgery technique. There was no statistically significant relationship between different techniques of surgery and the development of aphakic glaucoma (*p* = 0.33 for anterior vitrectomy; *p* = 1.00 for opening of the posterior capsular bag). No glaucoma was documented in the IOL-group; the *p* value for the relationship between those eyes who received an IOL at surgery and those who developed glaucoma was significant (*p* < 0.001).

During the follow-up period (median of 30.9 months, IQR: 20.8–62.8 months), 33 glaucoma surgeries were performed (number of surgeries): transconjunctival cyclophotocoagulation (27) and trabeculectomy (6). Three eyes underwent subsequent needling after trabeculectomy (twice in two eyes and five times in one eye).

At the last follow-up examination in the aphakic glaucoma group, 6/9 patients presented with strabismus and 7/9 patients with nystagmus. Visual acuity was ≥0.4 decimal Snellen in only one eye and ≤0.2 in three eyes, and two eyes had no light perception. No follow-up data concerning visual acuity were available for 6/12 eyes of this group.

## 4. Discussion

There was no association between the surgical procedure employed and the subsequent development of PCO, which was the most frequent postoperative complication. Our analysis of the large cohort confirmed aphakic glaucoma as a high-risk complication after congenital cataract surgery associated with ocular or syndromic anomalies. Nystagmus was diagnosed in a statistically significantly greater number of patients at last follow-up in the aphakic than in the IOL group.

### 4.1. Classification of Cataract

Congenital cataracts were the main reason for surgical intervention in our pediatric cataract group. The majority (68.3%) presented with isolated cataracts without associated anomalies, which is in accordance with previous reports [3, 17]. The presentation of bilateral cataract in 72.3% is in agreement with previous reports from different geographical areas [3, 4, 18, 19].

### 4.2. First Presentation and Examination

Analysis of the personal medical history revealed that 31.7% (20 patients) presented with extraocular and ocular anomalies or diseases. Syndromal congenital cataracts were most often bilateral (7/9). We had no cases of cataract due to congenital infections, contrary to other studies [4, 5]. This may be due to the high vaccination coverage and low incidence of diseases such as congenital rubella in Switzerland. The Federal Office of Public Health of Switzerland reported no cases of materno-fetal rubella between 2010 and 2016 [20]. PFV was present in five eyes with congenital unilateral cataract and in none with congenital bilateral cataract, which is in agreement with the study by Tartarella et al. [5]. They reported PFV in 8.2% of patients, and the majority of cases were diagnosed in patients with unilateral cataract [5].

### 4.3. Surgery

Median age at surgery was lower in the aphakic as compared to the IOL group. There is an ongoing controversy about the correct time of surgery due to the risk of developing aphakic glaucoma and amblyopia. Ruddle et al. reported earlier surgery as the “key risk factor” for the development of aphakic glaucoma [21], but Birch et al. state that a delay of surgery beyond six weeks was associated with a higher risk for strabismus and nystagmus [45]. A recently published retrospective study from Germany reported a strong relationship between cataract surgery within the first 14 weeks of life and the development of aphakic glaucoma [22]. They compared the outcome of bilateral congenital cataract treatment for those who underwent surgery within the first 10 weeks of life with those who underwent surgery between 10 weeks and 12 months of age [22]. The prevalence of amblyopia was not statistically different between the two age groups [22]. Nagamoto et al. concluded that glaucoma developed more frequently in eyes which required surgery at one year of age or younger [19]. The age at surgery for the aphakia group was 14 ± 24 months (mean ± SD), and their prevalence of aphakic glaucoma was observed to be 5.8% [19]. Chen et al. compared bilateral congenital cataract patients and observed a better outcome for those who underwent surgery at six months of age than for those at three months of age [23]. In our study, we observed glaucoma development in patients who underwent surgery at a very young age (in the first 4.7 months of age). There is no clear consensus in the literature regarding the best age for surgery to avoid development of glaucoma and amblyopia, even though some reports describe a better outcome for cataract surgery after the first three months of life [22, 23].

The microincision cataract surgery technique (23-gauge (23G)) was introduced in our institution in July 2013 and has been used for 12 cataract surgeries through to December 2014. According to Li et al., this technique has many advantages, particularly including fewer postoperative complications [10]. Lim et al. report that they applied transcorneal 23G-vitrectomy for routine congenital cataracts due to the small incision size, anterior chamber stability, and handpiece rigidity [24]. Given that our numbers and follow-up time for those who underwent surgery with the 23G-vitrectomy continue to be significantly limited, it is not yet possible to analyze data regarding functional outcomes.

### 4.4. Amblyopia Therapy

Amblyopia therapy was primarily performed in children with unilateral cataract. According to the Infant Aphakia Treatment Study, the number of reported hours of patching throughout the first four years of life is associated with a better visual acuity in cases of unilateral congenital cataracts [25]. Patching compliance was found to be one of the most important determinants of visual outcome [26]. Unfortunately, there was a lack of information within our reviewed medical charts regarding the daily hours of patching and the patients' compliance. Consequently, it was not possible to perform a detailed analysis of the visual outcome following amblyopia therapy.

### 4.5. Postoperative Complications

As shown in Figure 3, the most common postoperative complications were PCO (IOL group 38.8%, aphakic group 22.2%) and aphakic glaucoma, followed by contact lens-related corneal neovascularization and keratitis (both exclusively in the aphakic group) and synechiae (both groups). Bar-Sela et al. found a similar prevalence of PCO development with 38% in their patient group [27]. They concluded that the management of the posterior capsule at cataract surgery influences the incidence and the timing of PCO after IOL implantation [27]. We did not identify an association between the opening of the posterior bag or the performing of an anterior vitrectomy and the development of PCO. Retinal detachment occurred in 3.2% of the eyes, which is in agreement with a large previous study (3% of 1043 eyes) [28]. The variability in our follow-up period could lead to an underestimation of postoperative complications, especially in those patients with a short follow-up.

### 4.6. Aphakic Glaucoma

In accordance with previously published data from other tertiary centers, aphakic glaucoma is one of the main postoperative complications [7, 12, 29]. The incidence of aphakic glaucoma after cataract surgery at our institution is 12.8% of eyes and was diagnosed at a median time of 6.8 months after surgery. Our data is in agreement with other European studies, which reported a range of 9.7% to 15.3% incidence of developing aphakic glaucoma between 5.0 and 6.1 months after surgery [30, 31].

Aphakic glaucoma is often associated with abnormal ocular development such as microcornea [12], anterior segment dysgenesis, and aniridia [32, 33]. Microcornea is known to be an independent risk factor for aphakic glaucoma [7]. We were able to confirm this finding in our group with a significant relationship between the presence of microcornea and the development of glaucoma. There was an even stronger relationship between the presence of ocular developmental abnormalities and aphakic glaucoma. None of the patients who had experienced ocular trauma with consecutive cataract subsequently developed glaucoma.

At our institution, the median age at surgery of the aphakic glaucoma group was 45.0 days (mean 60.9 days), compared to the nonglaucoma group, which was 2.5 years. Other studies reported a slightly later onset of aphakic glaucoma in comparison to our group [29, 34]. A direct comparison may be problematic due to differing IOP criteria used to define glaucoma. According to Solebo et al., a tenfold higher age at the time of surgery decreases the development of glaucoma after cataract surgery by 60% and an older age at surgery is interpreted as a protective factor [12]. Ruddle et al. confirmed an association between younger age at time of surgery and a higher incidence of glaucoma [21]. This may explain the absence of glaucoma in the IOL group, considering that the median for age at surgery was considerably higher in the IOL group than in the aphakia group. Furthermore, the youngest child with an IOL implantation was 21 months old. In a randomized clinical control, Lambert et al. compared contact lens to intraocular lens correction of monocular aphakia in children who underwent surgery between one and six months of age. In their study, more children in the IOL group developed glaucoma by the age of one year [35].

Diagnosis of aphakic glaucoma was made at a median of 6.8 months postoperative, with the earliest onset being 11.0 days after surgery. These results are comparable to the British Congenital Cataract Study (median time 1.34 years; range of 12 days to 6.73 years) [36]. Our follow-up period (median 2.6 years, mean 3.4 years) is comparable to other European studies from 2.5 to 9.4 years [30, 31, 37, 38]. At the last documented follow-up examination, the majority of our patients presented with strabismus, nystagmus, and a poor visual outcome, which is in agreement with other studies [38]. The interpretation of the visual outcome in this group was limited by our retrospective study design with limited follow-up data.

The preferred treatment of congenital and early juvenile glaucoma at our department is trabeculotomy. We know from the recent literature that 360° trabeculotomy is preferable over standard trabeculotomy, for example, with the Harms probe [39]. Currently, we perform 360° trabeculotomy rather than standard trabeculotomy with the Harms probe. However, in some cases, the parents did not allow us to follow this standard treatment plan. In some cases, (1) examination under anesthesia (EUA) was performed in the children's hospital where it was not possible to perform ocular surgeries or (2) parents refused surgery on both eyes at the same time or refused immediate surgery after the EUA. In some cases, cyclodestructive procedures seem to be a valid option to lower the IOP until definite surgery can be performed.

### 4.7. Functional Outcome

To evaluate the functional and morphological outcome after cataract surgery, we considered visual acuity and the presence of strabismus and nystagmus at last follow-up.

#### 4.7.1. Visual Outcome

Median time of the last follow-up visit was 31.1 months postsurgery, which is comparable to other investigations [19, 40–42]. Similar to the results of other studies, we found development of visual acuity at both far and near distances to be more favorable in the bilateral cataract group [21] and for those individuals who had surgery at an age older than 2 years.

#### 4.7.2. Nystagmus

All patients who developed nystagmus in the follow-up period belonged to the aphakic glaucoma and bilateral cataract group (4/37 patients, 10.8%). We identified a statistically significant greater number of patients with nystagmus at last follow-up in the aphakic than in the IOL group. Furthermore, nystagmus was diagnosed more often in the bilateral than in the unilateral group at last follow-up, as also documented in other studies [18, 43, 44]. However, the small number of patients does not allow us to confirm a higher likelihood of nystagmus in children after bilateral than in unilateral cataract surgery. Nystagmus, and particularly latent nystagmus, may be underreported in the medical records, and the retrospective study design does not allow further analysis of this.

Preoperative nystagmus was no longer visible during the follow-up period in three patients, which was most likely caused by improved visual function. In a prospective investigation, Lloyd et al. described cases which converted postoperatively from preoperative nystagmus to manifest latent nystagmus (MLN) [6, 44]. Young et al. reported that preoperative nystagmus did not appear to be a risk factor for a worse visual outcome [11].

#### 4.7.3. Strabismus

There was no statistically significant difference regarding the incidence of strabismus that developed during the follow-up period between the unilateral and bilateral groups as well as between the IOL and aphakic groups. 19.0% (12/63) of patients were diagnosed with strabismus at first presentation. Tartarella et al. describe an even higher prevalence (52.2%) of strabismus preoperatively, but they did not report the postoperative data [5]. A study by Birch et al. investigated risk factors for strabismus development. They concluded that laterality and age at onset were significant nonmodifiable risk factors for strabismus [45]. A reduced risk for nystagmus and strabismus was associated with deprivation of less than 6 weeks [45].

### 4.8. Limitations of the Study

Our study represents a retrospective analysis of medical charts and surgical reports. Visual acuity analysis is depending on age and tests used. Preferable, only one method of visual acuity testing is used to compare data to avoid overestimating outcome data, especially when preferential-looking charts are used. This could not be achieved in this retrospective study but would encourage for further data collection in a prospective manner.

Data acquisition, especially of follow-up data, was partially restricted. Hence, there were no preestablished protocols regarding the timing and performance of surgical procedures and follow-up regimen. Furthermore, it is important to consider that differences in the age at surgery are influenced by the presentation and laterality of cataract. In 2012, the care of a number of aphakic patients was transferred to a private practice and therefore no clinical data was available after the point of transfer for these patients. The only factor in the transfer of these particular patients was parental desire to maintain continuity of care with the initially treating physician, rather than other demographic factors (e.g., age, outcome, and unilateral versus bilateral cataract), and so we agree that selection bias is highly unlikely to be introduced by this transfer. This data would have strengthened the statistical power of the follow-up data analysis, especially for visual development. Patients with medically uncontrolled glaucoma are always transferred back to our institution. Therefore, we are confident that all cases with severe aphakic glaucoma are included in our analysis, but cases with medically controlled aphakic glaucoma are likely to be underreported.

## 5. Conclusions

Congenital cataract was the main reason for surgical intervention in our pediatric cataract group, and most of the juvenile cataracts were classified as acquired. PCO was the most common postoperative complication and was observed significantly more often in the IOL group. There was no association between the surgical technique and the development of PCO postoperatively. Children who underwent surgery at an age older than two years and suffered from bilateral cataract had a better visual outcome. Aphakic glaucoma is the second most common postoperative complication and is often associated with ocular developmental abnormalities such as microcornea.

## Figures and Tables

**Table 1:** Classification of cataract.

*Congenital cataract*	82 eyes
No associated findings	56
Microcornea	12
Persistent fetal vasculature (PFV)	3
PFV and microphthalmia	1
PFV, microphthalmia, and posterior synechiae	1
Lentiglobus and lenticonus	2
Flat anterior chamber, protruding iris, and posterior synechiae	1
Abnormal greyish anterior sclera	2
Complex anterior segment dysgenesis	2
Aniridia (PAX6 mutation)	2

*Juvenile cataract*	12 eyes
Marden-Walker syndrome	2
Muscular dystrophy	2
Post laser coagulation for ROP	3
Juvenile rheumatic arthritis associated with chronic anterior uveitis	2
Posttraumatic cataract	2
Posttraumatic cataract and anterior lens capsule perforation	1

**Table 2:** Ocular anomalies and systemic diagnosis of the affected patients (20/63) with cataract are listed.

Ocular anomalies and systemic diagnosis		*N*
Complex ocular anomalies	Complex anterior segment dysgenesis (microcornea, posterior embryotoxon, posterior synechiae)	1
	PAX6 mutation^∗^ (aniridia, persistent tunica vasculosa lentis, macular hypoplasia)	1

Extraocular anomalies	Heart defects (microcornea *n* = 1^∗^)	4

Extraocular diseases	Juvenile rheumatic arthritis with chronic uveitis	1
	Nonclassified muscular dystrophy	1

Syndromes	Sakoda complex (agenesis of the corpus callosum and encephalocele, cleft palate, hypoplasia of the right optic nerve)	1
	Down syndrome (vitreous opacities *n* = 1)	2
	Dysmorphic syndromes of unknown origin (microcornea *n* = 1)	3
	Marden-Walker syndrome	1
	Lowe syndrome^∗^ (persistent tunica vasculosa lentis)	1
	Oculo-facio-cardio-dental syndrome	1

Varia	Prematurity of the newborn Gestational age: 31 weeks *n* = 2, 36 weeks *n* = 1 (laser scars due to the treatment of retinopathy of prematurity)	3

*N*: patient number. ^∗^Diagnosis of aphakic glaucoma during follow-up period.
